# Evaluating a large language model's ability to answer clinicians' requests for evidence summaries

**DOI:** 10.5195/jmla.2025.1985

**Published:** 2025-01-14

**Authors:** Mallory N. Blasingame, Taneya Y. Koonce, Annette M. Williams, Dario A. Giuse, Jing Su, Poppy A. Krump, Nunzia Bettinsoli Giuse

**Affiliations:** 1 mallory.n.blasingame@vumc.org, Information Scientist & Assistant Director for Evidence Provision, Center for Knowledge Management, Vanderbilt University Medical Center, Nashville, TN; 2 taneya.koonce@vumc.org, Deputy Director, Center for Knowledge Management, Vanderbilt University Medical Center, Nashville, TN; 3 annette.williams@vumc.org, Senior Information Scientist and Associate Director for Metadata Management, Center for Knowledge Management, Vanderbilt University Medical Center, Nashville, TN; 4 dario.giuse@vumc.org, Associate Professor, Department of Biomedical Informatics, Vanderbilt University School of Medicine and Vanderbilt University Medical Center, Nashville, TN; 5 jing.su@vumc.org, Senior Information Scientist, Center for Knowledge Management, Vanderbilt University Medical Center, Nashville, TN; 6 poppy.krump@vumc.org, Information Scientist, Center for Knowledge Management, Vanderbilt University Medical Center, Nashville, TN; 7 nunzia.giuse@vumc.org, Professor of Biomedical Informatics and Professor of Medicine; Vice President for Knowledge Management; and Director, Center for Knowledge Management, Vanderbilt University Medical Center, Nashville, TN

**Keywords:** Large Language Models, LLMs, Generative AI, Artificial Intelligence, Evidence Synthesis, Library Science, Information Science, Biomedical Informatics

## Abstract

**Objective:**

This study investigated the performance of a generative artificial intelligence (AI) tool using GPT-4 in answering clinical questions in comparison with medical librarians' gold-standard evidence syntheses.

**Methods:**

Questions were extracted from an in-house database of clinical evidence requests previously answered by medical librarians. Questions with multiple parts were subdivided into individual topics. A standardized prompt was developed using the COSTAR framework. Librarians submitted each question into aiChat, an internally managed chat tool using GPT-4, and recorded the responses. The summaries generated by aiChat were evaluated on whether they contained the critical elements used in the established gold-standard summary of the librarian. A subset of questions was randomly selected for verification of references provided by aiChat.

**Results:**

Of the 216 evaluated questions, aiChat's response was assessed as “correct” for 180 (83.3%) questions, “partially correct” for 35 (16.2%) questions, and “incorrect” for 1 (0.5%) question. No significant differences were observed in question ratings by question category (p=0.73). For a subset of 30% (n=66) of questions, 162 references were provided in the aiChat summaries, and 60 (37%) were confirmed as nonfabricated.

**Conclusions:**

Overall, the performance of a generative AI tool was promising. However, many included references could not be independently verified, and attempts were not made to assess whether any additional concepts introduced by aiChat were factually accurate. Thus, we envision this being the first of a series of investigations designed to further our understanding of how current and future versions of generative AI can be used and integrated into medical librarians' workflow.

## INTRODUCTION

Following the public launch of OpenAI's Chat Generative Pre-Trained Transformer (ChatGPT) in November 2022 [1], much consideration has been given in the academic and popular discourse to the current and anticipated impact of generative artificial intelligence (AI) on a number of professions. Within the health sciences, studies have investigated the ability of generative AI chat tools (including ChatGPT, Google Gemini, and Microsoft Copilot) to respond to patients' medical inquiries [2, 3], answer questions on licensing exams [4], support healthcare education [5], facilitate communication of research studies to lay audiences [6], aid with clinical documentation [7], and contribute to academic manuscripts [8], with many studies focused on specific specialty areas [4]. Much has also been written about the potential for librarians to become expert AI knowledge workers, who could play a critical role in advising and instructing library users on how to best use and integrate these AI tools for their information needs [9–12]. Although none of these studies were conducted in a medical library, many discuss its potential usefulness in that setting [10–16]. Outside the field of medical librarianship, Qureshi et al. [17] and Wang et al. [18] have explored how the application of generative AI could be developed to aid with search strategies and systematic reviews. None of the studies so far seem to have investigated the performance of generative AI in the critical task of searching and synthesizing knowledge from the medical literature, particularly in comparison with medical librarians' expertise in this area.

At the Center for Knowledge Management at Vanderbilt University Medical Center (VUMC), our team of medical librarians has, for over twenty years, provided evidence syntheses of the biomedical literature to respond to clinicians' questions, many of which are complex (i.e., questions containing clusters of questions), arising from clinical encounters. These questions were gathered initially through rounding with clinical teams and, since 2004, via an evidence request message basket service linked within the electronic health record (EHR) to facilitate clinicians' ability to send requests at the time and place when they most need an answer [19–23]. A previous study found high levels of physician satisfaction with the evidence summaries provided by our team [24]. This service requires librarians to be highly trained and able to quickly search and filter the current available literature on the topic, extract the most salient information needed to answer the question, and prepare a concise but comprehensive narrative synthesis that is returned to the clinician to inform decision-making [25]. Given the ability of generative AI chat tools to quickly produce detailed, fully articulated summaries drawn from a large body of knowledge, evaluating their current performance in responding to clinical questions is critical to understanding how they may eventually be integrated into medical librarians' workflows.

Some studies assessing generative AI tools' ability to provide comprehensive and accurate responses to clinical questions have observed that they can produce accurate results [26–29], particularly for less complex requests [26], although variation in results has been observed among different specialties, tasks, and models investigated [4]. Significant limitations have also been observed, including introduction of both minor and major errors via hallucination or misinterpretation [26, 30–31], lack of upto-date information [32], and limited domain-specific content knowledge [33]. However, with ongoing updates and refinement, it is anticipated that these tools will continue to improve, with advancements already observed, for example, in comparisons of GPT-3.5 to GPT-4 [26, 34].

Previous studies have evaluated generative AI chat bots' responses to clinical questions in comparison with a) published practice guidelines [27, 35–37], b) objective multiple-choice answers [4], and/or c) assessment by clinical experts' review [4, 26, 38–40]. Our study uses a different approach in that, to our knowledge, no studies have yet evaluated generative AI tools in the context of responding to actual clinical questions that arise from patient healthcare encounters and use medical librarians' evidence syntheses as a reference standard. This study builds upon previous research in knowledge acquisition [41–42] and continues our examination of how AI could aid or eventually transform medical librarians' work [43–45]. Given our institutional policies restricting the use of publicly available generative AI tools, we used aiChat [46], a VUMC-managed generative AI tool.

This study aimed to investigate the current ability of aiChat to answer individual clinical questions compared to expertly trained librarians when questions are formulated in a standardized manner. Specifically, the study investigated the following questions:

1. How accurate are aiChat's responses to clinical questions, as compared with medical librarians' gold-standard evidence syntheses?
2. Is aiChat's performance affected by question adjudication status (i.e., whether a third person was needed to resolve discordant ratings by two independent reviewers)?
3. Are there significant differences in aiChat's performance by question category?
4. What proportion of references included in aiChat responses can be verified to exist?

The main rationale for undertaking this study is to provide the field of biomedical librarianship with sufficient elements of investigation to promote interest and curiosity towards AI and its potential usefulness in our field.

## METHODS

Our team of information specialists has, for many years, received clinical questions from providers via rounding and via an evidence request message basket service linked within the electronic health record (EHR), providing evidence syntheses as a response. The evidence syntheses are created leveraging the comprehensive collection of journals at Vanderbilt University Medical Center, through freely available biomedical literature and grey literature published online, and via document delivery when needed. A sample of actual clinical questions was used to compare the accuracy of a locally managed generative AI chat tool's responses with librarians' gold-standard responses. Although these questions were generated by clinicians in connection to specific patient cases, they do not, by design, include any identifiable patient information, and the study was determined to be exempt by the Vanderbilt University Medical Center Institutional Review Board (IRB 240714). As applicable, this study adhered to the JAMA Network Guidance for Reporting Use of AI in Research and Scholarly Publication [47].

### Generative AI Tool

As submission of proprietary data to public-facing generative AI tools is restricted by our medical center policy, we used an organizationally approved, internally managed AI chat tool called aiChat to conduct the study [46]. aiChat is an in-house instance of OpenAI's GPT-4 large language model made available to users at our institution behind a secure firewall using Microsoft Azure's cloud computing services [48]. At the time of the study, aiChat was a Beta version with options to use either OpenAI's GPT-3.5 or GPT-4 models. Similar to the public version of ChatGPT, aiChat allows users to choose the GPT model of interest, submit one or more prompts, and receive a response in a user-friendly, conversational format. For this study, we chose to use the GPT-4 model due to the improvements in accuracy, advanced reasoning, and its more extensive training data set compared to GPT 3.5 [49–50]. The model was used as provided by aiChat; no additional dataset was used to train the algorithm.

### Question Pool

The in-house database used to assign, document, and archive clinicians' questions and the corresponding evidence synthesis responses provided by our team was queried to retrieve all questions received since 2010 [20, 21, 23, 51]. To align with GPT-4's most recent knowledge cutoff date at the time of the study, we excluded questions received after April 2023. A group of information scientists then determined eligibility of each archived question. The question set was limited to those that addressed a clinician's information need during patient care; general education questions and patient education requests were excluded. Questions were also excluded if the evidence synthesis response provided by the librarian contained just a list of citations, provided only an annotated list of citations, or reported that no answer was found in the literature. Prior to being assigned the task of establishing eligibility, all librarians worked on the same sample set of questions to determine consistency. As the eligibility criteria were clearly defined and easy to understand, the reviewers were quickly able to resolve the few minor differences in interpretation.

For this initial study, we aimed to assess aiChat's performance when responding to one focused question at a time, with future analyses planned to assess aiChat's performance with entire complex, multi-faceted requests comprised of clusters of questions. Therefore, requests containing more than one distinct question (e.g., both diagnosis and treatment) were divided into individual questions by information scientists in alignment with the methods established by Giuse et al. [52]. Each was considered a separate question for the study. In some cases, questions were reworded for clarity or to remove irrelevant information from the requestor's original message (e.g., details about requested turnaround time).

### Determining Critical Elements of the Responses

The librarians' original evidence summaries were used as the gold standard for comparison with aiChat's response summaries. To facilitate the comparison, pairs of medical librarians reviewed the original evidence synthesis response for each included question and came to consensus on the concepts that were most critical (i.e., the part of text directly answering the question at hand) and necessary to answer each question. For this process, librarians focused on the most pertinent, high-level conclusions, in recognition that there may be wide variation in wording and other elements within narrative summaries that nonetheless reach the same conclusions. These critical elements were copied from the original response and recorded in REDCap [53, 54] alongside the question, prior to submitting the question to aiChat.

### Prompt Engineering and Submission

**Figure 1:**
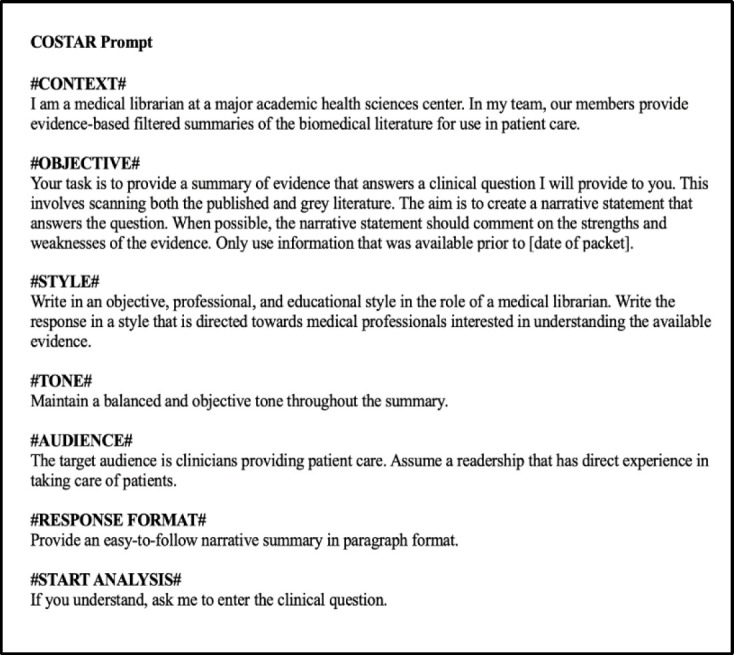
Standardized prompt, in COSTAR format, used to submit each question to aiChat.

Consultation of the literature for current practices for effective prompt engineering revealed no widely accepted, authoritative guidelines. However, researchers have suggested approaches to improve the quality of generative AI's response, which were consistent with observations from initial testing by our team, such as giving the chat bot a clear role, establishing the context, and defining the expected output in terms of format and audience [55–57]. The COSTAR framework (Context, Objective, Style, Tone, Audience and Response) [58] was selected to guide prompt engineering for this study as it provides specific details to inform the GPT response, including the use of delimiters to specify the input's distinct components, and incorporates many of the principles recommended in the literature [58–61]. Using the framework, senior members of the team with expertise in librarianship, knowledge acquisition, medicine, and artificial intelligence devised a standardized prompt to submit with each clinical question (Figure 1).

COSTAR=Context, Objective, Style, Tone, Audience and Response [58].

OpenAI's GPT-4's training data set includes a variety of Internet sources, including books, articles, and websites; specific details are generally not available [62–63]. aiChat was prompted to only use data from its training set published prior to the date of the original clinician request for evidence to avoid inclusion of information that would have not yet been published, and thus not available to the librarian compiling the evidence summary. In testing, aiChat was able to adjust the response by date when given this parameter. Given that studies have established that GPT often fabricates references [14, 30, 64], the team did not specifically ask aiChat to provide references as part of the prompt. Providing an example of the desired output within the prompt has also been suggested [61] and found to improve performance in some analyses [18]. However, it is unlikely that a user asking a real clinical question would have an example response readily available to submit, so examples were not included in our prompt.

All questions were submitted to aiChat between March 25, 2024 – April 1, 2024. To capture aiChat's responses to each question, medical librarians worked in pairs to submit assigned sets of questions to the chat bot tool. First, a librarian selected “New prompt,” set aiChat to use GPT-4, and submitted the prompt (Figure 1). When aiChat responded to confirm understanding (e.g., “Understood. Please enter the clinical question.”), the clinical question was copied directly from the REDCap database and submitted within the same encounter. The full response from aiChat was copied from the interface and saved in REDCap.

Initially, a set of five randomly selected test questions was submitted to aiChat five times each back-to-back in sequence by a senior member of our team to assess whether there was enough variation in the responses to necessitate submitting each question multiple times. Although variance was observed in the wording and other elements of aiChat's summary replies, the overall concepts and conclusions were consistent. Other research has observed significant differences in ChatGPT's responses when prompts are submitted multiple times [18]. However, this study aimed to assess the performance of generative AI in the real-life scenario of a clinician seeking a response to a clinical question. In this context, submitting a question multiple times would not be practical. Thus, for this study, the team decided to submit each question only one time.

### GPT Response Evaluation

Each question, along with the critical elements from the original packet and the response from aiChat, was assigned to two independent medical librarian reviewers to evaluate the extent to which aiChat's response aligned with the original librarian's gold-standard synthesis of evidence from the published and grey literature. Each reviewer independently assessed whether aiChat answered the question correctly in comparison with the original gold-standard response. The assessment was based on whether aiChat included all, some, or none of the key critical elements that were identified by consensus from the librarian's original summary. Reviewers used a 3-point Likert scale adapted from Suárez et al. [40] to indicate whether aiChat's overall response was incorrect (1), partially correct (2), or correct (3). See Table 1 for detailed descriptions of each grading. The response options avoid the use of non-numerical, vague qualitative terminology (e.g., “mostly correct”), as these types of phrases may create ambiguity and difficulty with interpretation [65–66]. For example, different reviewers may interpret and apply the concept of “mostly” in different ways. In our study, the determination of partially correct (2) was quantitatively determined by counting the number of absent critical elements (Table 1). To be considered correct, it was not necessary for aiChat to use the exact same language from the original summary, but rather for the response to be conceptually similar. In cases where aiChat provided information beyond what the librarian included, the responses were not considered incorrect. If the aiChat summary included facts not present in the librarian's summary, no efforts were made to assess their accuracy.

**Table 1:** 3-point Likert scale score criteria for GPT response evaluation.

Reviewer's grading	Description
**Incorrect (1)**	The answer does not address any critical elements identified in the librarian's original evidence summary.
**Partially Correct (2)**	The answer addresses one or more, but not all, critical elements identified by medical librarians.
**Correct (3)**	The answer addresses all critical elements identified in the original evidence summary.

Adapted from Suárez et al. [40]

For example, one question included in the study asked for recommendations on hyponatremia treatment with free water restrictions and sodium chloride (NaCl) tablets. The critical elements identified from the librarian's gold-standard response were: 1. Guidelines suggested the use of oral NaCl combined with loop diuretics for syndrome of inappropriate antidiuretic hormone secretion (SIADH) patients with hyponatremia; 2. Guidelines also recommended fluid restriction and hypertonic saline use for hyponatremia; 3. Some recommendations also addressed fluid restriction for SIADH and other conditions without mentioning the use of NaCl tablets. Overall, the aiChat answer addressed all key elements and was categorized as correct (3). If one or two of the three critical elements had been absent, this answer would have been classified as partially correct (2). The absence of all critical elements would make the answer incorrect (1).

### Adjudication of Discordant Ratings

Discordant ratings of aiChat's performance by the two independent librarian reviewers were resolved (adjudicated) by a third reviewer with medical knowledge and expertise in evidence synthesis, librarianship, knowledge acquisition, and extensive experience with adjudication in knowledge acquisition research [67–68]. The adjudicator thoroughly reviewed each question, the full original summary, the complete aiChat summary, and, if needed, the original supporting references. When relevant, association websites referred to by the aiChat tool were also consulted. Specifically, we consulted websites as they existed at the time the original question was asked by using the Internet Archive's Wayback Machine [69]. This process allowed us to confirm whether more recent knowledge that would not have been available to the librarian at the time of the original request may have been incorporated into aiChat's response and thus created discrepancies.

### Question Categories

To allow comparison of performance by question type, each question was assigned by a medical librarian to one of eight distinct categories: Disease Etiology, Diagnostic Procedure, Differential Diagnosis, Disease Description, Disease Complication, Disease Prevention, Disease Prognosis, or Treatment. These categories were adapted from a previous study conducted by NBG [52, 70]. In cases of multi-faceted questions, each of the individual questions was assigned a category.

### Reference Verification

Although the prompt did not specifically request the inclusion of references, many of aiChat's responses did contain academic references with combinations of author name, journal, and/or publication year. The assessment of accuracy was based only on aiChat's summary. A separate exploratory analysis was performed using a sub-sample of questions to verify if the references provided by aiChat were real or hallucinated.

For this analysis, a smaller sample of questions with responses that included citations was identified through random selection using a random number generator [71]; each question was assigned to a pair of librarians. The librarians reviewed the responses from aiChat and independently attempted to locate all cited references using the details provided (e.g., author name, article title, publication date). Our team searched PubMed, Google, Google Scholar, and journal websites to verify whether the references supplied by aiChat could be matched to a published source. We documented whether the citation was found or not found, and, if located, assessed its open access status. A single librarian checked whether the located references were cited in the original evidence summary packet.

### Statistical Analysis

The ratings for all questions were stored in REDCap and analyzed descriptively using medians, ranges, and frequency. For each question, the absolute (n) and relative frequency (%) of ratings of incorrect (1), partially correct (2), and correct (3) were tabulated. For group comparisons of the categorical data, we used Wilcoxon Rank Sum, Kruskal-Wallis, or Fisher's Exact tests. The Wilcoxon Rank Sum test was used for the analysis of nonparametric ordinal ratings when compared across two independent groups. The Kruskal-Wallis test was used for analysis of nonparametric ordinal ratings when compared across more than two independent groups, and Fisher's Exact test was used to evaluate nominal ratings across more than two independent groups All analyses were conducted with GraphPad Prism 10 software. A two-tailed p-value <0.05 was used as the threshold for statistical significance.

## RESULTS

The study included 217 discrete questions. During adjudication, one question was excluded due to misclassification as a patient care-related question. The final number of questions analyzed for the study was 216.

### Evaluation of the Accuracy of aiChat's Responses

Table 2 shows the overall ratings of the accuracy of the tool's responses compared with the medical librarians' gold-standard evidence syntheses. Overall, 180 (83.3%) of aiChat responses were assessed as correct in comparison with the original librarian's response, while 35 (16.2%) were assessed as partially correct and 1 (0.5%) was assessed as incorrect.

**Table 2:** Ratings of aiChat's responses to discrete questions, by adjudication status.

Questions	Incorrect (1)	Partially Correct (2)	Correct (3)	Total
Questions without adjudication	1 (0.5%)	28 (15.4%)	153 (84.1%)	182 (84.3%)
Questions with adjudication	0 (0.0%)	7 (20.6%)	27 (79.4%)	34 (15.7%)
**Total**	**1 (0.5%)**	**35 (16.2%)**	**180 (83.3%)**	**216 (100%)**

### Performance by Adjudication Status

Consensus was achieved between librarian pairs on 182 (84.3%) of the responses; the remaining 34 (15.7%) responses required adjudication. Results were similar for responses requiring and not requiring adjudication, with 84.1% (n=153) of questions without adjudication and 79.4% (n=27) of questions with adjudication assessed as correct. The Wilcoxon Rank Sum test revealed there were no statistically significant differences in the ratings of responses that received adjudication in comparison to those that did not undergo adjudication (p=0.61). Of the adjudicated questions, most (n=32) were due to a discrepancy of one point (e.g., scores of partially correct [2] and correct [3]). Two questions were adjudicated due to a discrepancy between incorrect (1) and correct (3) scores.

### Comparison by Question Category

The most common question category was Treatment (n=147; 68.1%), which included topics such as treatment adverse effects and treatment efficacy, while the least commonly assigned category was Differential Diagnosis (n=1; 0.46%). The percent of aiChat responses assessed as correct was ≥80% across all categories. No significant differences were observed in the question ratings by category when evaluated by the Kruskal-Wallis test (p=0.73), nor were any patterns or trends identified. For a full report of results by each category, see Table 3.

**Table 3:** Ratings of aiChat's responses to discrete questions, by question category.

Question Category	Number of Questions	Incorrect (1)	Partially Correct (2)	Correct (3)
Disease Etiology	20	1 (5.0%)	1 (5.0%)	18 (90.0%)
Diagnostic Procedure	10	0 (0.0%)	2 (20.0%)	8 (80.0%)
Differential Diagnosis	1	0 (0.0%)	0 (0.0%)	1 (100%)
Disease Description	10	0 (0.0%)	1 (10.0%)	9 (90.0%)
Disease Complication	8	0 (0.0%)	0 (0.0%)	8 (100%)
Disease Prevention	7	0 (0.0%)	1 (14.3%)	6 (85.7%)
Disease Prognosis	13	0 (0.0%)	1 (7.7%)	12 (92.3%)
Treatment*	147	0 (0.0%)	29 (19.7%)	118 (80.3%)
**Total**	**216**	**1 (0.5%)**	**35 (16.2%)**	**180 (83.3%)**

* aggregates the treatment, treatment adverse effects, and treatment efficacy question categories

### Comparison by Adjudication and Question Category

The questions sent for adjudication at the highest proportion were related to disease prevention (n=2; 28.6%); none of the differential diagnosis questions were adjudicated (Table 4). No patterns were observed in the data, and there were no significant differences by category of questions that received adjudication when compared by Fisher's Exact test to questions that were not adjudicated (p=0.90).

**Table 4:** Adjudication status of aiChat's responses to discrete questions, by question category.

Question Category	Number of Questions	No Adjudication	Adjudication
Diagnosis Etiology	20	18 (90.0%)	2 (10.0%)
Diagnostic Procedure	10	8 (80.0%)	2 (20.0%)
Differential Diagnosis	1	1 (100%)	0 (0%)
Disease Description	10	9 (90.0%)	1 (10.0%)
Disease Complication	8	7 (87.5%)	1 (12.5%)
Disease Prevention	7	5 (71.4%)	2 (28.6%)
Disease Prognosis	13	12 (92.3%)	1 (7.7%)
Treatment*	147	122 (83.0%)	25 (17.0%)
**Total**	**216**	**182 (84.2%)**	**34 (15.8%)**

* aggregates the treatment, treatment adverse effects, and treatment efficacy question categories

### Verification of References from GPT Response

Though the prompt we used to submit the clinical questions to aiChat did not specifically ask citations to be included, the responses provided by the GPT often did include references. Sixty-six (30%) answers which included 162 references were randomly selected for citation verification. The number of references provided by aiChat per question ranged from 1–4, with a median of 2.45. Our team was able to verify the existence of 60 of the 162 references (37.0%). Most of the verifiable citations were indexed in PubMed (n=56; 93.3%), with the remaining available on the cited journal's website (n=2; 3.3%), a professional organization's website (n=1;1.67%) and the website of the Food and Drug Administration (n=1;1.67%). Of these 60 references, 35 were open access. Nineteen references (31.7%), all open access, overlapped with some of the citations used by the librarians in answering 14 questions.

## DISCUSSION

In this initial study comparing generative AI summaries with medical librarians' gold-standard clinical evidence syntheses in response to individual clinical questions, an organizationally managed generative AI chat tool using GPT-4 was able to report key elements identified in the librarian's evidence synthesis for the majority of clinical questions examined. These results are promising but only a first step in what we foresee to be a series of many investigations into generative AI tools' ability to summarize the evidence to answer clinical questions. We recognize the complexity and responsibility of creating a valid, comprehensive, and trustworthy evidence synthesis and are cognizant of many of the issues discussed in an article from Zhang and colleagues, including the need to ensure that large language models are trustworthy, transparent, secure, and avoid perpetuating biases [72].

In our sample of clinical questions, aiChat provided a correct response for 83.3% of questions and a partially correct response for 16.2%, resulting in an overall 99.5% of questions having at least a partially correct response. Most of the questions in our study (68.1%) were treatment-related, which is consistent with the types of questions most frequently asked by clinicians [52, 70, 73]. No significant differences in accuracy were observed across different categories of clinical questions or adjudication status. The one summary rated by the reviewers as incorrect was a response to a question about genetic mutations associated with a particular disease, for which aiChat's response referenced a different gene than the one reported in the gold-standard evidence packet. This finding could possibly suggest a need to better understand how generative AI tools handle genetic information given the complexity of the field.

While the aiChat- and medical librarian-developed summaries were consistent overall in terms of the key concepts included, many (63%) of the supporting references included in a subsample of aiChat's responses could not be independently verified. The inability to trust references provided by large language models and, consequently, to be able to verify specific details and results of the studies cited in the responses they provide is currently a significant limitation to their use. However, it is possible that generative AI tools' performance in this area could improve as we continue to see a rise in open access publishing [72, 74–75] and the models are not as limited by subscription paywalls. Furthermore, the wider availability of open access resources may make it easier to fully trace and verify the sources underlying generative AI's responses [62–63]. Issues of copyright are also well-discussed in the literature; this remains a key issue, as researchers are largely unable to determine the full set of content used to train the large language models [32, 76–78]. Although it is important to note that in some instances, the information in the response could be drawn entirely from freely available abstracts, the lack of transparency on the details of the datasets still poses real concerns.

We also anticipate that GPT may improve its response if provided with a curated set of full-text articles selected by a medical librarian. The ability of AI tools to allow users to enter content could greatly improve the very controversial and troubling problem of reference hallucination [14, 30, 64]. Providing a new generation of generative AI tools with selected content may also aid in addressing ethical concerns when using large language models, which reflect the social biases and inequities present in the clinical research studies and other content included in their training sets [72, 79–80]. By selecting content to provide to the generative AI tool, we could additionally ensure that copyright issues are addressed [32, 76–78] and that content with curated references is fully representative of a diverse population and as free as possible from bias.

Tang et al. [31] conducted a study using ChatGPT and GPT-3.5 in which the generative AI tools were provided with content from Cochrane review abstracts from six clinical areas and prompted to provide four-sentence summaries of the systematic reviews. The study found that, in this context, the summaries included few instances of fabrication; however, errors (e.g., those related to misinterpretation of the content) were still observed. In November 2023, OpenAI introduced a feature allowing users to create custom GPTs through which they can provide their own knowledge (e.g., full-text articles or other written documents) for GPT to use when responding to prompts [81]. At the time of the study, this feature was not available through our organization's internal generative AI tool, but OpenAI does offer the ability to create custom GPTs at the Enterprise level to enable organizations to leverage this option with proprietary information. Tools harnessing generative AI to search and summarize academic papers using underlying literature databases (e.g., Consensus [82] and Scopus AI [83]) are also becoming available. Additional studies are needed in this area to fully understand current models' ability to accurately summarize research when provided with selected, full-text source material.

In addition to assessing generative AI tools' performance relative to that of humans, Shah and colleagues have also emphasized the importance of evaluating the benefits of large language models and considering how they can be leveraged to enhance our work rather than simply replicating it [84]. In this study, we observed that a strength of the aiChat responses was the formatting of the narrative summaries, which typically began with a brief introduction to the topic, followed by a well-organized summary with a balanced representation of the viewpoints found in the literature, and ended with brief conclusions. While the requestor receiving the evidence synthesis may be an expert who is already familiar with the topic, they may also wish to share the summary to educate other members of the team with varying specialties (e.g., pharmacists, nutritionists) or who may be more junior (e.g., medical students). Our team recognizes that the approach of establishing the background at the beginning of the response has educational value in our academic setting and considers the inclusion of all viewpoints in the literature to be a best practice for evidence synthesis [70, 85]. The organization used by aiChat to structure the responses also has educational value for our profession as a model that can be applied for instructional purposes to train clinical librarians.

A review article by Lund et al. [13] on how librarians in different fields and specialties could incorporate generative AI in their work also reports on interesting opportunities for use in medical librarianship, like using the AI tool as a digital assistant and research partner in a variety of information specialist roles. Mughari and colleagues [16] more specifically adds the potential for AI to be used by librarians in collection development, digital content curation, indexing, and data analytics, among other things. Sutton and Parisi [14], Roth and Wermer-Colan [15], and Friesen et al. [11] see generative AI as playing a role in searching and the systematic review process. Liu and colleagues [10], Zhang [9], Friesen et al. [11], and Epstein [12] additionally discuss how AI technologies represent a new opportunity for user instruction and training by librarians. It is undoubtedly the case that having librarians, in their role of educated knowledge workers, become active players in this AI revolution could provide them with a truly transformative way of incorporating this new technology in their profession.

## LIMITATIONS

An assumption of this study was that medical librarians' original evidence syntheses accurately reflected the literature as of the original request date, and that clinicians who received the response trusted and agreed that the supporting evidence provided by the librarian answered their questions. Although we did not independently reverify the information provided in these evidence syntheses, previous studies have found high levels of physician satisfaction with our team's evidence services [24].

Similarly, we did not assess the accuracy of every detail of aiChat's summary but rather focused on whether the most critical elements of the librarian's original response were present and, for a subset of questions, whether references could be verified to exist. No attempts were made in this study to evaluate whether any additional facts introduced by aiChat were accurate or whether the verified references were cited appropriately, as the comparison was based on whether the critical elements identified in the librarian's gold-standard response were included in aiChat's answer.

It is important to note, that this study was conducted at a single large academic medical center, and that may be reflected in the type/complexity of questions included in our dataset. Thus, the study may not be entirely generalizable to other environments.

For this study, we intentionally divided complex, multi-faceted questions (i.e., a question which includes a cluster of questions) into individual questions and separately evaluated aiChat's response to each question. aiChat's performance in responding to complex, multi-faceted questions taken as a whole was not evaluated.

Finally, it is possible that aiChat's performance was impacted by elements of prompt design, such as the lack of examples in the prompt or our decision to only submit each question once.

## CONCLUSIONS

The findings of this study highlight the promising performance of a generative AI tool using GPT-4 for providing responses to individual clinical questions, while also confirming known limitations, such as reference fabrication. Our approach is replicable for other institutions who may wish to conduct similar investigations, but it would not apply to institutions where organizational policies do not allow the use of publicly available generative AI tools and do not provide their own internal versions. Since the aim of this study was to evaluate whether aiChat was able to answer clinical questions with a response which included the answer given by our established gold standard, we intentionally did not evaluate any additional conceptual differences in the summaries.

Additional avenues for future research include exploring generative AI's ability to respond to questions for which librarians found no answer and evaluating aiChat's answers to multi-faceted clinical questions. We also plan to conduct research 1) evaluating performance across multiple versions of GPT models to understand how they continue to evolve and improve over time, and 2) investigating which of the generative AI usage recommendations listed in our discussion above could most successfully be carried out in our environment. Given the current inability to independently verify many of the sources used for the generative AI responses, an important next step will be to conduct a more detailed analysis of the source material. A particular area of interest is to establish a better understanding of the extent to which questions can be answered through freely available open source literature. It will also be critical to understand how generative AI performance may improve when provided with a body of literature curated by expert medical librarians. This model could potentially couple GPT's strengths in summary generation with librarians' critical expertise in literature selection and assessment.

## Data Availability

The clinical questions used in this study are not publicly available as the data is institutional proprietary information.
